# Genome-Wide Characterization and Anthocyanin-Related Expression Analysis of the *B-BOX* Gene Family in *Capsicum annuum* L.

**DOI:** 10.3389/fgene.2022.847328

**Published:** 2022-02-28

**Authors:** Jin Wang, Guangbin Yang, Ying Chen, Yao Dai, Qiaoling Yuan, Qingyun Shan, Luzhao Pan, Li Dai, Xuexiao Zou, Feng Liu, Cheng Xiong

**Affiliations:** ^1^ College of Horticulture, Hunan Agricultural University, Changsha, China; ^2^ College of Horticulture, Nanjing Agricultural University, Nanjing, China; ^3^ Hunan Vegetable Research Institute, Hunan Academy of Agricultural Sciences, Changsha, China; ^4^ Engineering Research Center for Horticultural Crop Germplasm Creation and New Variety Breeding, Ministry of Education, Changsha, China

**Keywords:** pepper, CaBBXs, duplication, development expression, anthocyanin biosynthesis

## Abstract

The transcription factors, B-box (BBX), belong to a subfamily of the zinc finger family of proteins and exhibit multiple biological functions in plant growth, development, and abiotic stress response pathways. In this study, a total of 23 *CaBBX* members were identified using the pepper reference genome database. According to the gene structure, conserved domains, and the phylogenetic tree, 23 *CaBBX* genes were divided into four groups, wherein the analysis of the promoter region indicated the presence of *cis*-acting elements related to plant development, hormones, and stress response. Interspecies collinearity analysis showed that the *CaBBXs* had three duplicated gene pairs, and the highest gene density was found on chromosomes 2 and 7. Transcriptome RNA-seq data and quantitative polymerase chain reaction (qRT-PCR) analysis of pepper plants spanning the entire period showed that more than half of the *CaBBX* genes were widely expressed in diversity tissues of pepper. Co-expression network analysis indicated that the *CaBBXs* and the anthocyanin structural genes had a close co-expression relationship. Thus, it was reasonably speculated that the *CaBBX* genes may be involved in the regulation of anthocyanin biosynthesis. Overall, this study involved the genome-wide characterization of the *CaBBX* family and may serve as a solid foundation for further investigations on *CaBBX* genes involved in the anthocyanin synthesis mechanisms and development in pepper.

## Introduction

Pepper (*Capsicum*), an important species of the Solanaceae family, has significant popularity and economic value. It is widely cultivated in almost all areas with arable land, owing to its high nutritional value and complex germplasm diversity (Qin et al., 2014). As an important trait in pepper, the color is a vital indicator for classification, maturity, and evolutionary characteristics of the pepper species (Liu et al., 2020a). A previous study shows that anthocyanins, carotenoids, and beet pigments are the main factors that determine the color of leaves, flowers, and fruits in natural plants and that their types and contents control the shades of different organs (Tang et al., 2020).

Anthocyanins, a flavonoid compound, is widely present in plants. They can not only impart color and attract pollinators and seed dispersers but also protect them from ultraviolet radiation. Anthocyanins play an important regulatory role in several plants through their antioxidants, phytoales, and antibacterial effects (Tang et al., 2020). Peppers rich in anthocyanins can reduce the risk of cancer, alleviate neurological dysfunction, and prevent cardiovascular diseases (Fang, 2015). The biosynthesis of anthocyanins is characterized by catalysis through an enzymatic cascade. First, malonyl-CoA and coumarin-CoA are used as substrates to produce chalcone through the catalysis of chalcone synthase (CHS). Chalcone is catalyzed by chalcone isomerase (CHI) to produce naringenin, which is then converted to dihydrokaempferol by flavanone 3-hydroxylase (F3H). Dihydroflavonol 4-reductase (DFR), UDP-glucose: flavonoid 3-glucosyltransferase (UFGT), and anthocyanin synthase (ANS) regulate the anthocyanin synthesis in different species. Anthocyanin reductase (ANR) and leucoanthocyanidin reductase (LAR) are the key factors in proanthocyanidin biosynthesis (Alappat and Alappat, 2020). The synthesis of various enzymes regulates the biosynthesis of anthocyanins.

The biosynthesis of anthocyanins is also regulated by several transcription factors. Among them, the MYB-bHLH-WD40 ternary complex (MBW complex) as a typical representative is known to positively regulate anthocyanin biosynthesis. In *Arabidopsis*, the WBM complex includes four *MYB* transcription factors, namely *AtPAP1*, *AtPAP2*, *AtMYB113*, and *AtMYB114*, three *bHLH* genes, *AtGL3*, *AtEGL3,* and *AtTT8*, and a typical WD40 protein, AtTTG1. In apple, the *MdMYB10* transcription factor was first identified as a member of the MBW complex and found to induce the accumulation of anthocyanins in heterologous and homologous species. It is positively correlated with anthocyanin levels during fruit development in apple plants. In carrot (*Daucus carota*), the *DcMYB7* gens regulate the inheritance of anthocyanin pigmentation in purple and non-purple carrot roots (Xu et al., 2019). In the subtropical deciduous tree species, Formosan sweetgum (*Liquidambar formosana* Hance), the *R2R3-MYB* transcription factor gene, *LfMYB113* is positively correlated with anthocyanin content in the leaves (Wen and Chu, 2017). In transgenic tomato fruits, the overexpression of two MYB genes, *SlANT1* or *SlAN2,* results in the accumulation of anthocyanins and the up-regulation of *EBGs*, *LBGs,* and *SlAN1*. The bHLH protein, SlANT1, influences the induction of anthocyanin biosynthesis (Meng et al., 2015). In pepper, the *CaAN2* gene is the main transcription factor that regulates and controls anthocyanin biosynthesis; the variations in the promoter non-LTR retrotransposon region caused the differences in colors of the pepper fruits (Jung et al., 2019). Simultaneously, the *CaAN2* gene can regulate the expression of structural genes (*CaF3′5′H*, *CaDFR*, *CaUFGT*, *CaCHS*, and *CaF3H*) and modulate the anthocyanin content in pepper (Zhang et al., 2015). In addition to the MBW complex that directly regulates anthocyanin biosynthesis, some other transcription factors also play a regulatory role in anthocyanin synthesis, directly or indirectly. For example, in petunia, the *WRKY* transcription factor, *PhPH3,* and MBW complex regulate the expression of the two proton pumps (PhPH1 and PhPH5) that are further responsible for vacuolar acidification, thereby imparting the red color to the anthocyanins (Verweij et al., 2016). An anthocyanin biosynthesis gene, *Ca3GT*, has been finely located in the pepper chromosome 10, and it is a strong candidate gene for anthocyanin biosynthesis in mature and immature pepper fruits (Liu J. et al., 2020).

In addition, environmental stresses, including extreme light intensity, mechanical damage, and/or low temperature can induce the accumulation of anthocyanins through the MBW complex and regulation by other transcription factors (Movahed et al., 2016). Light is an important environmental factor influencing the accumulation of anthocyanins. High light intensity stimulates the gene expression and production of anthocyanins in many species (Su et al., 2016). Among them, the B-box (BBX) zinc finger protein family transcription factors have attracted much attention in recent years. The N-terminus of the BBX proteins contain one or two conserved BBX domains (B-box1 and B-box2), and some members also possess a CCT (Constans, CO-like, and TOC1) structure at the C-terminal region (Huang et al., 2012). Several studies show that under light-mediated conditions, the B-box (BBX) protein interacts with *ELONGATED HYPOCOTYL 5* (*HY5*) and *CONSTITUTIVE PHOTOMORPHOGENIC 1* (*COP1*) and further, mediates their regulatory activities (Gangappa and Botto, 2016). In rice (*Oryza sativa*), anthocyanin biosynthesis is induced and fine-tuned by *OsBBX14* and *OsHY5*. The ectopic expression of *OsBBX14* in *Arabidopsis thaliana* results in a significant increase in the accumulation of anthocyanins in its seedlings (Kim et al., 2018). In apple, the BBX protein MdBBX37 plays a negative role in light signal transduction. *MdBBX37* inhibits the binding of *MdMYB1* and *MdMYB9*, two key positive regulators of anthocyanin biosynthesis, with their target genes, thereby contributing to the negative regulation of anthocyanin biosynthesis pathway (An et al., 2019). The expression of *PpBBX16* in pear (*Pyrus pyrifolia*) is highly induced by white light irradiation, and the transient overexpression of *PpBBX16* in the pear skin increases the accumulation of anthocyanins, while the virus-induced *PpBBX16* gene silencing reverses this trend substantially (Bai et al., 2019). In poplars (*Populus L.* spp.), *BBX23* directly binds to the promoter regions of proanthocyanidins and anthocyanin-specific genes and interacts with HY5 to enhance their activation (Li et al., 2021).

Although BBX-mediated flavonoid accumulation has been reported in multiple species, whether the candidate *CaBBX*s in pepper were related to anthocyanins, remains unknown. In this study, the *CaBBX* family were identified in pepper from the draft of the whole genome sequences, and the *CaBBX* family of genes in pepper were found to have a wide range of biological functions in the fruit development processes, with divergent regulatory effects in the regulatory processes of anthocyanin synthesis. In conclusion, this finding may provide central guidance for future research on the function of *CaBBX* genes in peppers, and laid a solid foundation for the study of the biosynthesis mechanism of pepper anthocyanins.

## Materials and Methods

### Identification of *CaBBX* Transcription Factors in Pepper

To identify the BBX family of transcription factors, the hidden Markov model (HMM) profile of the B-box-type zinc finger domain (PF00643) was downloaded from the Pfam database (El-Gebali et al., 2019). The reference genomes of hot pepper, *C. annuum* cv. CM334 (Criollo de Morelos 334), and a Chinese inbred derivative, ‘Zunla-1’, were used in this study. The HMMER SEARCH3.0 with cutoff E value ≤0.01 and SMART database were used to confirm the conserved domains among the members of the BBX family (Finn et al., 2011). Theoretical pI and Mw values were computed using the ExPaSy online tool (Gasteiger et al., 2003). The SWISS-MODEL online tool was used for protein modeling (Schwede et al., 2003), and the softberry online software was used to predict the putative subcellular localization of genes (http://www.softberry.com).

### Conserved Motifs, Gene Structures, and Phylogenetic Analysis

The conserved domains were estimated using the MEME online tool (Version 5.1.0, National Institutes of Health, Bethesda, MD, United States) and these domains were aligned using DNAMAN (Version 8.0.8, Lynnon Biosoft). The Gene Structure Display Server was used to predict the gene structures of *CaBBXs* (Hu et al., 2015). The full-length amino acid sequences of *CaBBXs* were acquired from the pepper reference genome, “Zunla-1” (http://peppersequence.genomics.cn/), the sequences of SlBBXs were obtained from the tomato reference genome “SL4.0” (https://solgenomics.net/organism/Solanum_lycopersicum/genome), and those of AtBBXs were obtained from the *Arabidopsis* reference genome (https://www.arabidopsis.org/). MEGA-X was used to construct an unrooted neighbor-joining phylogenetic tree of CaBBXs, SlBBXs, and AtBBXs proteins with the bootstrap test replicated 1,000 times (Kumar et al., 2018).

### Synteny Analysis and *Cis*-Elements

The intra-species collinearity analysis of the *CaBBXs* was performed with MCScanx (Wang et al., 2012). The synonymous (Ks) and nonsynonymous (Ka) substitution rates were estimated using the KaKs_Calculator1.2 (Zhang et al., 2006). The Circos diagram showed the chromosomal location, collinearity, and Ka/Ks values for the genes (Krzywinski et al., 2009). 2.0 kbp upstream sequences of CDS were selected as the promoter region, and the PlantCARE web tool was used to predict the *cis*-elements in each gene (Lescot et al., 2002). The Tbtools were used for plotting (Chen et al., 2020).

### Materials, and RNA-seq Analysis

The TransZol kit (TransGen Biotech, Inc., Beijing, China) was used to extract total RNA, and HiScript^®^ⅡQRT SuperMix for qPCR (+gDNA wiper) vazyme kit (Vazyme, Piscataway, NJ, United States) was used to synthesize the reverse cDNA. The raw data for the RNA-seq analysis were downloaded from Pepper Hub (Liu et al., 2017), and a high-generation inbred capsicum line “6421”, was used. Fastqc was used for quality control of the sequencing data (Brown et al., 2017) and the low-quality sequences were removed using Trimmmatic-0.36 (Bolger et al., 2014). HISAT2 was used to align the sequencing reads to the reference genome, “Zunla” (Kim et al., 2014), and FeatureCounts was used to calculate the number of counts (Liao et al., 2014). Standardize counts data from DESeq2 package in R (Varet et al., 2016), and the fragments per kilobase of exon model per million mapped reads (FPKM) value represented the corresponding gene expression.

### Co-Expression Analysis

The weighted Gene Co-Expression Network Analysis (WGCNA) was used to analyze the FPKM value from RNA-seq data. The WGCNA package in R v3.6.1 language was used for the analysis (Langfelder and Horvath, 2008). The TOM similarity algorithm calculated the adjacent ordered function formed by the gene network and the coefficient of difference between the different nodes; the weighted gene correlation network analysis method was used to analyze the gene expression patterns in multiple samples. The co-expression correlation matrix was calculated and the gene expression correlation in the network was estimated. Cytoscape v3.6.0 was used to draw the correlation network diagram for the non-weight coefficients (weights) of the relevant *CaBBXs* and anthocyanin structural genes in the extracted matrix (Shannon et al., 2003).

### GO Enrichment Analysis and Real-Time Fluorescence Quantitative PCR

GO enrichment analyses were conducted using Plant Transcriptional Regulatory Map (Jin et al., 2017) and WEGO2.0 (Ye et al., 2006) online tool with the corrected *p*-value <0.05. LightCycle * 96 Real-Time PCR System (Roche, Basel, Switzerland) was used for performing qRT-PCR. A 50 μl reaction system was set and three biological repeats and three technical repeats were performed for each sample based on HiScript^®^ⅡQ RT SuperMix for qPCR (+gDNA wiper) vazyme kit (Vazyme, Piscataway, NJ, United States). The 2^−△△Ct^ formula was used to calculate the relative expressions. The primers for *CaBBXs* and *actin* control (*Capana04g001698*) were listed in Supplementary Table S2.

## Results

### Genome-Wide Identification and Conserved Domains of *CaBBX* Genes

According to the hidden Markov model (HMM)-based profile (Bbox-type zinc-finger domain, PF00643), the genome-wide annotated proteins were screened and verified using both SMART and Pfam databases. A total of 23 *CaBBX* genes were identified in the pepper Zunla-1 genome, however, 22 *CaBBXs* were present in the pepper CM334 genome. Based on their conserved motifs, 23 *CaBBX* genes were named as *CaBBX1* to *CaBBX23* within the gene subfamilies. The length of the coding sequences ranged between 564 (*CaBBX23*) to 1476 (*CaBBX22*) amino acid residues, while their molecular weights (Mw) varied greatly between 20.33293 KDa (*CaBBX23*) and 54.98339 KDa (*CaBBX22*). The isoelectric points (PI) of the *CaBBXs* ranged between 4.29 (*CaBBX23*) and 9.17 (*CaBBX21*), and most of them were weakly acidic proteins. Prediction software for subcellular localization suggested that these CaBBX proteins were mainly localized in the nucleus; five members, including *CaBBX7*, *CaBBX19*, *CaBBX21*, *CaBBX22*, and *CaBBX23* were found to localize to extracellular structures, such as chloroplasts and mitochondria (Table 1).

**TABLE 1:** The information on the *CaBBX* gene family.

GeneID	Zunla_2.0	CM334_1.55	Chr	Start	End	Strain	PI	Mw	Subcellular localization
*CaBBX1*	*Capana02g002620*	*CA00g70490*	Chr02	148180402	148183481	+	8.47	22972.52	Nuclear
*CaBBX2*	*Capana04g000266*	*CA04g21130*	Chr04	4091269	4092722	−	6.24	33860.88	Nuclear
*CaBBX3*	*Capana06g000735*	*CA06g21770*	Chr06	11697317	11699646	−	5.00	25979.56	Nuclear
*CaBBX4*	*Capana07g001114*	*CA00g25500*	Chr07	154056816	154057654	+	5.03	24831.96	Nuclear
*CaBBX5*	*Capana07g002062*	*CA07g16670*	Chr07	212563126	212570473	+	4.98	32012.77	Nuclear
*CaBBX6*	*Capana08g002611*	*CA01g19640*	Chr08	149745414	149746937	+	5.62	29831.52	Nuclear
*CaBBX7*	*Capana08g002625*	*CA01g19780*	Chr08	149926925	149929248	−	6.17	23515.68	Extracellular
*CaBBX8*	*Capana09g000394*	*CA07g16670*	Chr09	12877150	12887068	+	4.97	32173.20	Nuclear
*CaBBX9*	*Capana11g002294*	*CA11g01070*	Chr11	218148220	218150401	−	4.85	35202.56	Nuclear
*CaBBX10*	*Capana12g000659*	*CA12g17630*	Chr12	18213299	18215385	−	6.50	35220.55	Nuclear
*CaBBX11*	*Capana00g001486*	*CA11g11000*	Chr00	399610115	399614902	−	6.75	51670.91	Nuclear
*CaBBX12*	*Capana01g004030*	*CA01g28430*	Chr01	278243944	278245872	−	5.45	37932.28	Nuclear
*CaBBX13*	*Capana02g003199*	*CA02g26670*	Chr02	157107846	157110095	+	5.41	44556.35	Nuclear
*CaBBX14*	*Capana02g003200*	*CA02g26680*	Chr02	157118313	157120062	+	5.27	43889.44	Nuclear
*CaBBX15*	*Capana02g003201*	*CA02g26690*	Chr02	157124787	157126407	+	5.32	45394.43	Nuclear
*CaBBX16*	*Capana03g000377*	*CA03g33660*	Chr03	5273465	5275489	−	5.53	43464.09	Nuclear
*CaBBX17*	*Capana03g003558*	*CA03g08890*	Chr03	228734754	228738294	−	5.45	43342.99	Nuclear
*CaBBX18*	*Capana07g000030*		Chr07	1563552	1565426	−	6.56	42540.99	Nuclear
*CaBBX19*	*Capana07g001588*	*CA07g12780*	Chr07	191773542	191774577	+	4.60	27399.14	Extracellular
*CaBBX20*	*Capana12g000414*	*CA12g19270*	Chr12	8179392	8181909	−	5.29	39425.86	Nuclear
*CaBBX21*	*Capana00g004911*	*CA06g15580*	Chr00	672862631	672863374	−	9.17	27712.80	Extracellular
*CaBBX22*	*Capana05g001195*	*CA05g08470*	Chr05	84622805	84628926	+	6.00	54983.39	Extracellular
*CaBBX23*	*Capana10g000048*	*CA10g00340*	Chr10	574455	577401	−	4.29	20332.93	Extracellular

**FIGURE 1:**
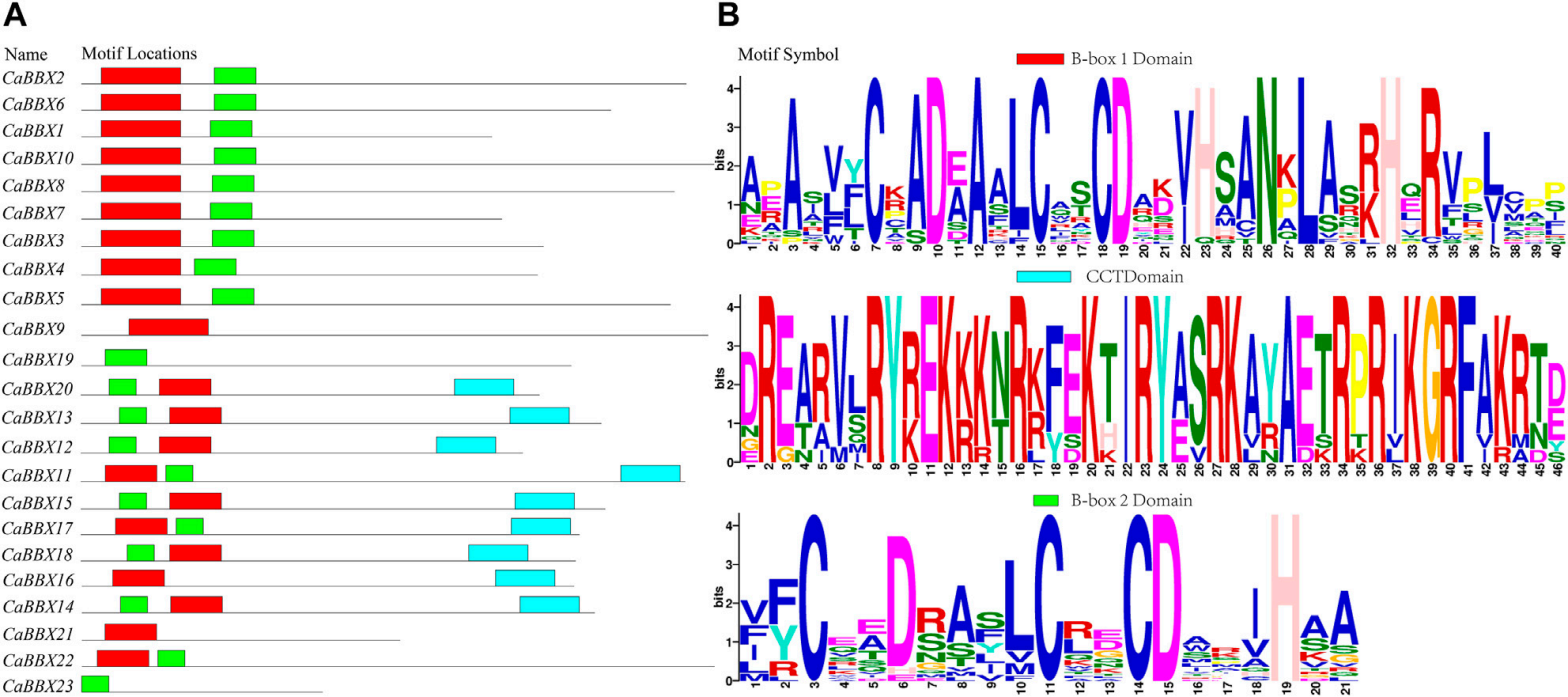
The domains of the *CaBBX* gene family. **(A)** The distributions of conserved motifs in *CaBBXs*. **(B)** Typical logo of the *CaBBX* conserved domains.

The conserved sequence of the zinc finger domain of B-box was found to exist in two forms, namely B-box1: C-X_2_-C-X_7–8_-C-X_2_-D-X-A-X-L-C-X_2_-C-D-X_3_-H-X_2_ -N-X_4_-H (indicated in red color), and B-box2 of C-X_2_-C-X_8_-C-X_7_-C-X_2_- C-X_4_-H(N)-X_6–8_-H (indicated in green color). Protein sequence alignment showed that the two B-BOX domains had highly similar conserved sequences (Supplementary Figure S1). For example, the cysteine (C) and aspartic acid (D) residues constituting the zinc fingers were highly conserved in the B-BOX domain. However, the conservative motif symbol, as shown in Figure 1B, was reversed owing to the conserved amino acid residues (Asn, Leu, His, and Arg) in the B-box1 domain; the B-box1 domain motifs were more conserved than those of the B-box2 domain. In addition, the BBX family also had a highly conserved CCT domain R-X_5_-R-Y-X_2_-K-X_3_-R-X_3_-K-X_2_-R-Y-X_2_-R-K-X_2_-A-X_2_-R-X-R-X_2_-G-R-F-X-K, as shown as a blue bar in Figure 1A. Among all the 23 CaBBX proteins, only three members (*CaBBX*9, *CaBBX*16, *CaBBX*21) contained one B-box1 domain, while two genes (*CaBBX*19, *CaBBX*23) had only one B-box2 domain. The remaining 18 genes existed in both two B-box domains, and nine members contained the CCT-conserved domain.

### Phylogenetic Relationships, Gene Structure, and *Cis*-Elements in the CaBBX Family

**FIGURE 2:**
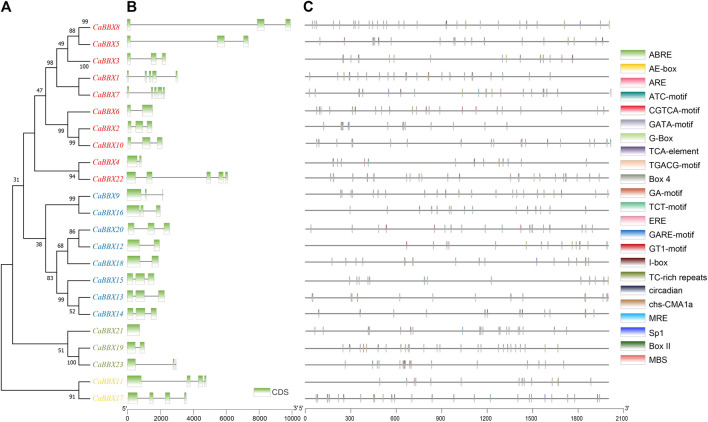
Phylogenetic relationships, gene structure analysis, and predicted *cis*-elements of *CaBBXs*. **(A)** Phylogenetic tree for the 23 *CaBBXs*. Red genes were classified in Group I, blue members belonged to Group II, green genes to Group III, and yellow members to Group IV. **(B)** Green boxes represent the coding sequence (CDS) regions while intron regions are indicated by black lines. Exon lengths can be inferred from the scale at the bottom. **(C)** Promoter sequences (−2,000 bp) of *CaBBX*s were analyzed using PlantCARE. Different shapes and colors represent different elements. Annotated *cis*-elements are listed in Supplementary Table S1.

Based on conserved sequences in the proteins, all the 23 CaBBX proteins were categorized into five distinct phylogenetic classes. In evolutionary classification, CaBBX1, CaBBX2, CaBBX3, CaBBX5, CaBBX6, CaBBX7, CaBBX8, CaBBX10, CaBBX4, and CaBBX22 genes were found to be homologous (Group I), and they all contained two conserved B-box domains. CaBBX9, CaBBX16, CaBBX20, CaBBX12, CaBBX18, CaBBX15, CaBBX13, and CaBBX14 genes were in the Group II. CaBBX21, CaBBX19, and CaBBX23 were classified as Group III, while Group IV included CaBBX11 and CaBBX17 proteins (Figure 2A). The gene structure analysis indicated that the number of exons in the *CaBBX* family of genes in pepper was between one and five. Among them, 12 (52.17%) *CaBBXs* had three exons, five (21.74%) *CaBBXs* had two exons, two *CaBBXs* had four exons, and three *CaBBXs* had five exons. *CaBBX21* only had one exon (Figure 2B). To examine the phylogenetic relationship of the *CaBBX* gene family, a neighbor-joining (NJ) phylogenetic tree was constructed based on the CaBBX protein sequences in pepper, AtBBX protein sequences in Arabidopsis, and SlBBX protein sequences in tomato. According to the phylogenetic tree, all BBXs were divided into four clades, wherein, each clade contained a similar number of BBX genes from the three species. Almost all these four clades corresponded to their designated domains, while some CaBBX proteins with similar domains (such as CaBBX2 in Clade II and CaBBX6 in Clade III) were clustered into different clades (Supplementary Figure S2).

2,000 base pair (bp)-upstream of the coding region was selected as the promoter sequence in the *CBBXs* to predict the *cis*-regulatory elements. A total of three *cis*-element types were identified in the promoter regions of the *CaBBox* gene family. A few genes had *cis*-elements related to cellular development, such as flavonoid biosynthetic genes regulatory elements. In addition, many CaBBX genes contained *cis*-elements related to stress response pathways, including responsiveness to light, anaerobic induction, defense and stress responsiveness, and circadian control. Six typical hormone-related *cis*-regulatory elements in the promoter regions were identified, including abscisic acid response elements, salicylic acid, and gibberellin, and MeJA response elements. Specifically, there were 13 *cis*-elements for light responsiveness, including Box 4, G-Box, TCT-motif, GT1-motif, GATA-motif, I-box, AE-box, chs-CMA1a, Sp1, ATC-motif, MRE, GA-motif, and Box II (Figure 2C).

### Chromosomal Distribution and Interspecies Synteny Analyses for CaBBXs

**FIGURE 3:**
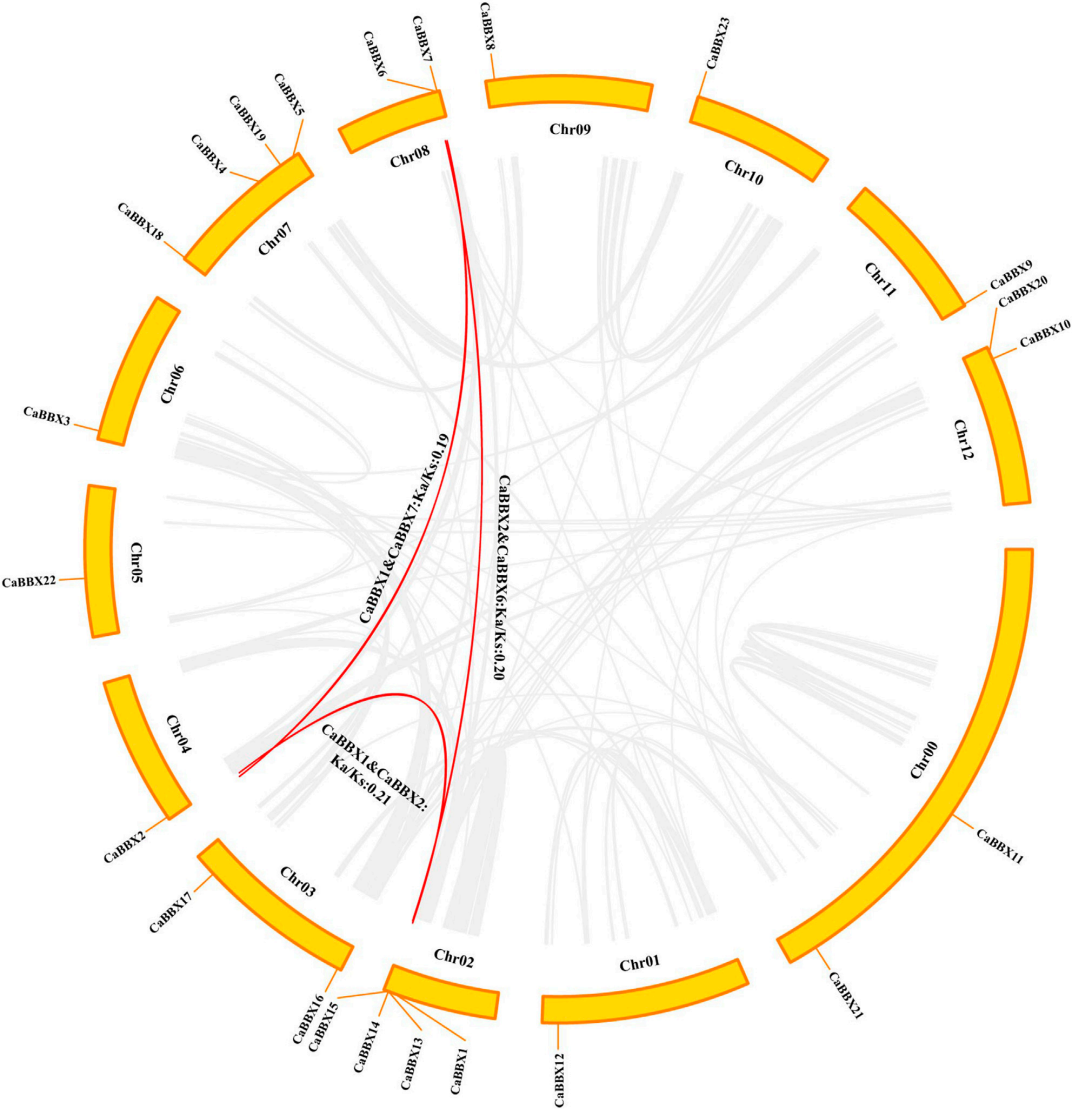
Chromosomal location and interspecies gene synteny in *CaBBX*s. The chromosomal positions of *CaBBX* genes in the pepper genome. Red lines in the middle indicate duplicated gene pairs of *CaBBX*s. The positions of *CaBBX* genes in the pepper genome are marked on chromosomes; Chr refers to chromosomes.

According to the physical positional information for the CaBBX gene family in the annotated zunla-genome, the 23 CaBBXs were found to be widely distributed in all 12 chromosomes, “01 g” to “12 g”, of pepper. Two CaBBX genes (CaBBX11 and CaBBX21) were located on chromosome “00”. Chromosomes 7 and 12 contained the most number of CaBBX genes, each harboring four CaBBXs. Chromosomes 8, 12, and 13 each contained two genes, and the remaining chromosomes each harbored one CaBBX member (Figure 3).

Only three segmental duplicated pairs and no tandem duplicates were observed for CaBBXs in pepper. In particular, CaBBX1-CaBBX2 and CaBBX2-CaBBX6 were pairs of mutually duplicated genes, while CaBBX1-CaBBX7 were present as duplicates. CaBBX6 and CaBBX7 were located on the same chromosome and were in close physical proximity. The selection pressure in duplicated genes was estimated using the values of non-synonymous mutation (Ka), synonymous mutation (Ks), and their ratio (Ka/Ks). The Ks values ranged between 1.76 and 3.16. The Ka/Ks values for all duplicated gene pairs were less than 1.00 and between 0.128 and 0.5. It was reversed that the three groups of CaBBX duplicated genes underwent purifying selection during the process of evolution. The minimum Ks and maximum Ka/Ks values were between the duplicate gene pair, CaBBX1-CaBBX2, and these two genes may have undergone greater purifying selection (Figure 3).

### Expressions of CaBBX DEGs in Developmental Stages Analyzed by RNA-seq

**FIGURE 4:**
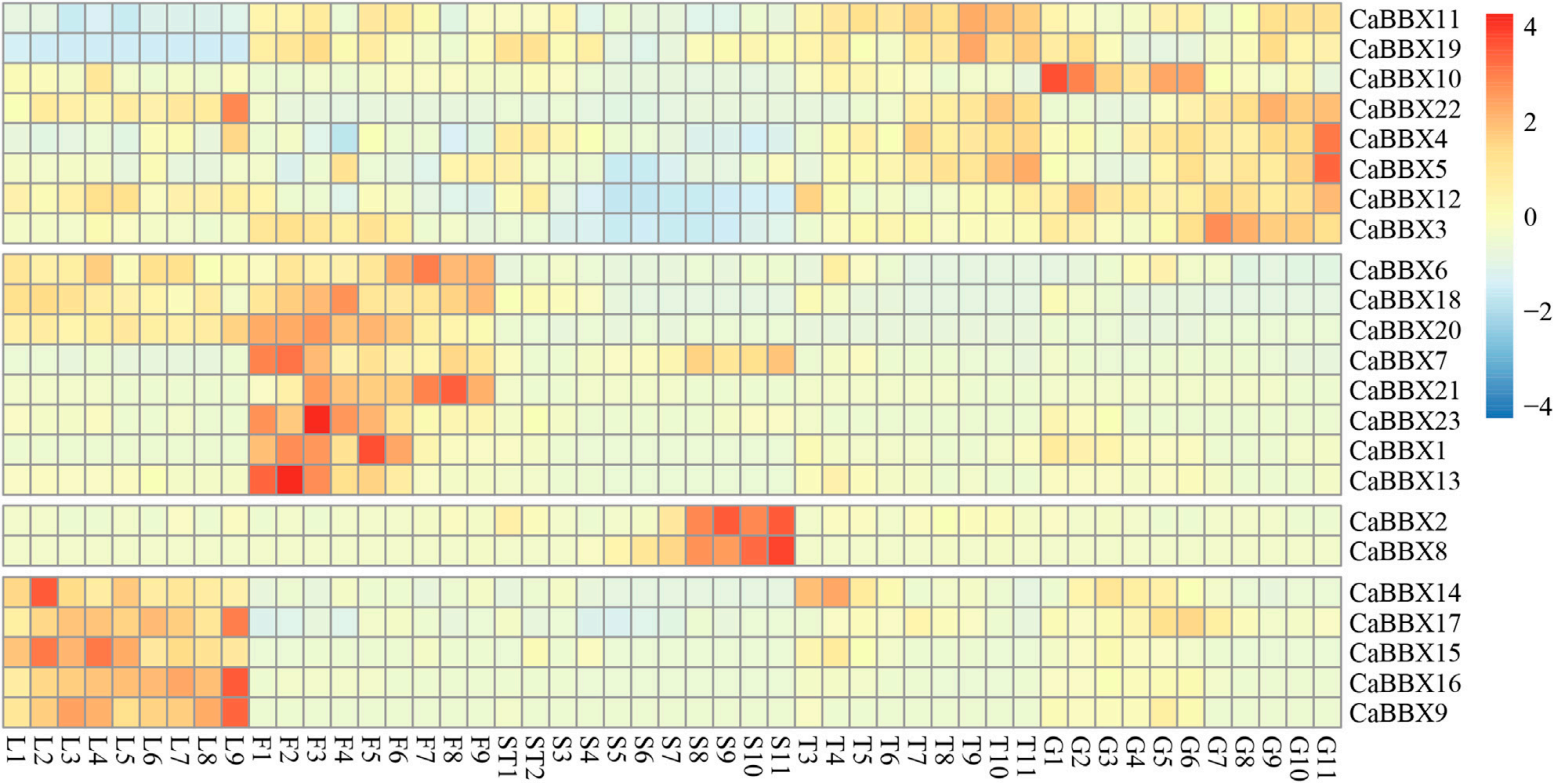
A Expression patterns of *CaBBXs*. Expression profiles using RNA-seq fragments per kilobase million (FPKM) data in the leaves between ten DAP and 60 DAP (L1–L9), flowers between ten DAP and 50 DAP (F1-F9), placenta and seed between ten DAP and 15 DAP (ST1 and ST2), the placenta between 20 DAP and 60 DAP (T3–T10), and pericarp between ten DAP and 60 DAP (G1–G11).

To gain insight into the potential functions of the CaBBX gene family spanning the entire developmental stage of the pepper plant, the raw BAM data of RNA-seq for various organs and tissues at different developmental stages were downloaded from the Pepperhub online database and used for analyzing the expression profiles. As shown in Figure 4, most CaBBX genes showed differential expressions in the different organs and tissues. Based on the differential tissue expressions, the 23 CaBBXs were divided into five groups. Among them, five CaBBXs (CaBBX14, CaBBX15, CaBBX16, CaBBX17, and CaBBX9) were relatively highly expressed in the leaves, while CaBBX2 and CaBBX8 were specifically highly expressed in the late seed stages and were not expressed or showed low expression in other tissue types. CaBBX22, CaBBX5, CaBBX4, CaBBX3, CaBBX10, and CaBBX12 showed high expression in both placenta and fruit, and CaBBX6, CaBBX18, CaBBX20, CaBBX7, CaBBX21, CaBBX23, CaBBX1, and CaBBX13 were highly expressed in all the flowering stages. Specifically, CaBBX11 and CaBBX19 showed low levels of expression across all the stages in leaf development, while CaBBX12 and CaBBX3 showed low levels of expression across all the stages of seed development. These results indicated that the CaBBX genes had diverse functions during the growth and development of pepper plants.

### GO Enrichment and Co-Expression of CaBBXs and Anthocyanin Structural Genes

**FIGURE 5:**
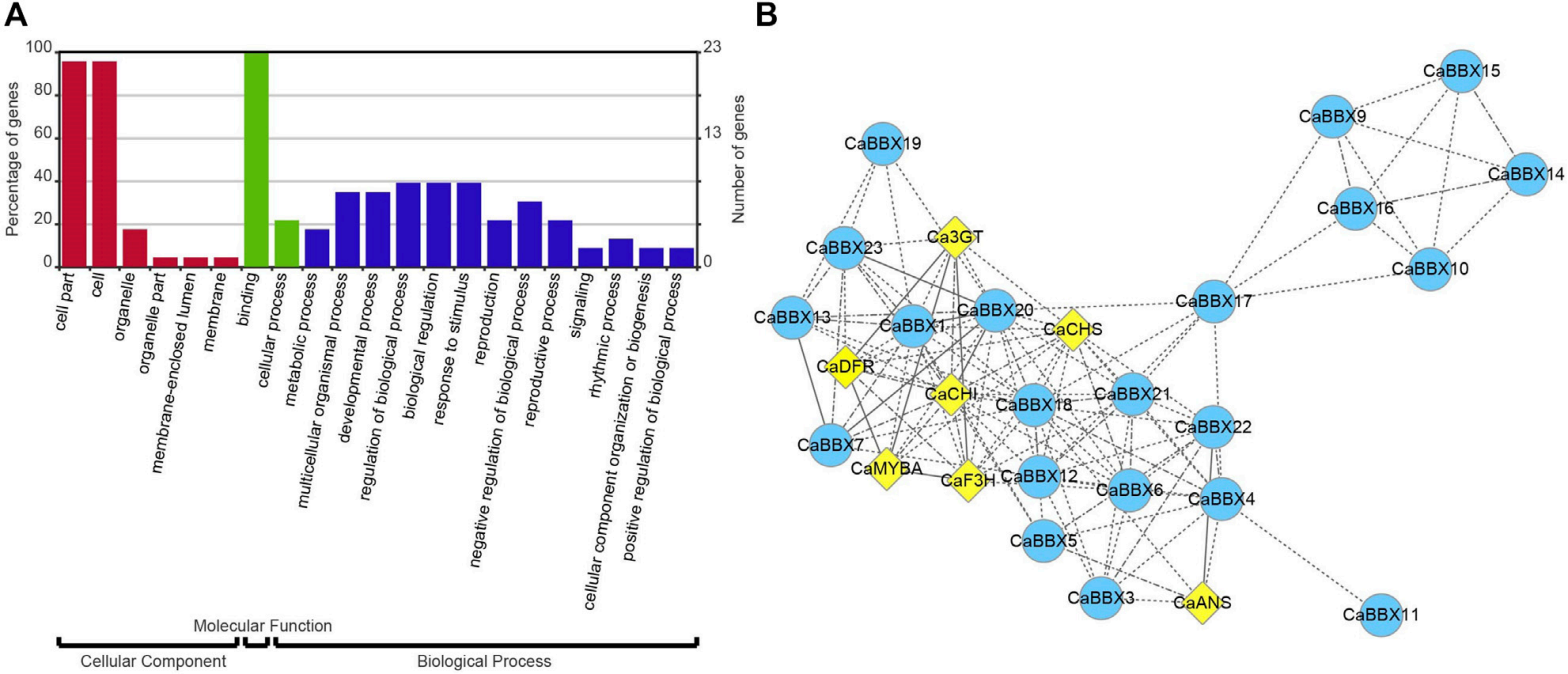
**(A)** GO enrichment analysis of *CaBBXs*. The red bars represent cellular component, the green bars represent molecular function, and the blue bars represent biological process. **(B)** Co-expression analysis of *CaBBXs* and structural genes of the anthocyanin pathway. The yellow diamonds represent the structural genes of the anthocyanin pathway and the blue circles represent the *CaBBX* family of genes. The line indicates their correlation; the more solid is the line, the higher is the correlation.

All 23 *CaBBXs* were used enrichment analysis and conducted on the assigned GO terms with the corrected *p*-value <0.05. Three GO categories were assigned, including cellular component, molecular function, and biological process. In cellular component, the cell part and cell were highly enriched in 23 *CaBBX* genes. In molecular function, *CaBBX*s were assigned in binding and cellular process. In biological process, *CaBBX*s were widely assigned in metabolic process, multicellular organismal process, developmental process, regulation of biological process, biological regulation, response to stimulus, reproduction, negative regulation of biological process, reproductive process, signaling, rhythmic process, cellular component organization or biogenesis, and positive regulation of biological process (Figure 5A).

According to the expression levels of 23 *CaBBXs* in different organs, the candidate *CaBBX* genes were predicted related to the anthocyanin synthesis pathway according to tissue specific expression. As shown in Figure 5B, all *CaBBX* genes were used for the construction of the co-expression network, along with key structural genes in the anthocyanin synthesis pathway. Among them, seven *CaBBX* members (*CaBBX1*, *CaBBX9*, *CaBBX13*, *CaBBX16*, *CaBBX20*, *CaBBX21*, and *CaBBX23*) (weighted TOM value >0.05) were mostly co-expressed with anthocyanin biosynthesis. In the early anthocyanin synthesis stages, *CaCHS*, and *CaCHI* were highly co-expressed with *CaBBX1*, *CaBBX20*, *CaBBX21*, and *CaBBX23*. *CaF3H* were co-expressed in the network but showed low co-expression with the genes in the anthocyanin synthesis pathway. In the late anthocyanin synthesis stages, *CaDFR* was also highly co-expressed with *CaBBX1*, *CaBBX20*, *CaBBX21*. And *CaANS* was highly co-expressed with *CaBBX9*, *CaBBX15*, and *CaBBX16*. Besides, *R2R3-MYB* transcription factors *CaAN1* and *CaAN2*, which involved in anthocyanin biosynthesis were also co-expressed with *CaBBXs*. *CaBBX1*, *CaBBX20*, and *CaBBX21* were co-expressed with *CaAN2,* but *CaBBX9*, *CaBBX15*, and *CaBBX16* were co-expressed with *CaAN1.* In co-expression network, *CaBBX3*, *CaBBX4*, *CaBBX5*, *CaBBX6*, *CaBBX7*, *CaBBX11*, *CaBBX14*, *CaBBX15*, *CaBBX17*, *CaBBX18*, and *CaBBX29* were indirect co-expression with key seven *CaBBX* genes or anthocyanin structural genes to affect anthocyanin biosynthesis. In addition, there was a close co-expression relationship between the four *CaBBX* genes (*CaBBX2*, *CaBBX8*, *CaBBX12*, and *CaBBX22*).

### qRT-PCR Analysis for Expressions of CaBBXs and Anthocyanin Structural Genes

**FIGURE 6:**
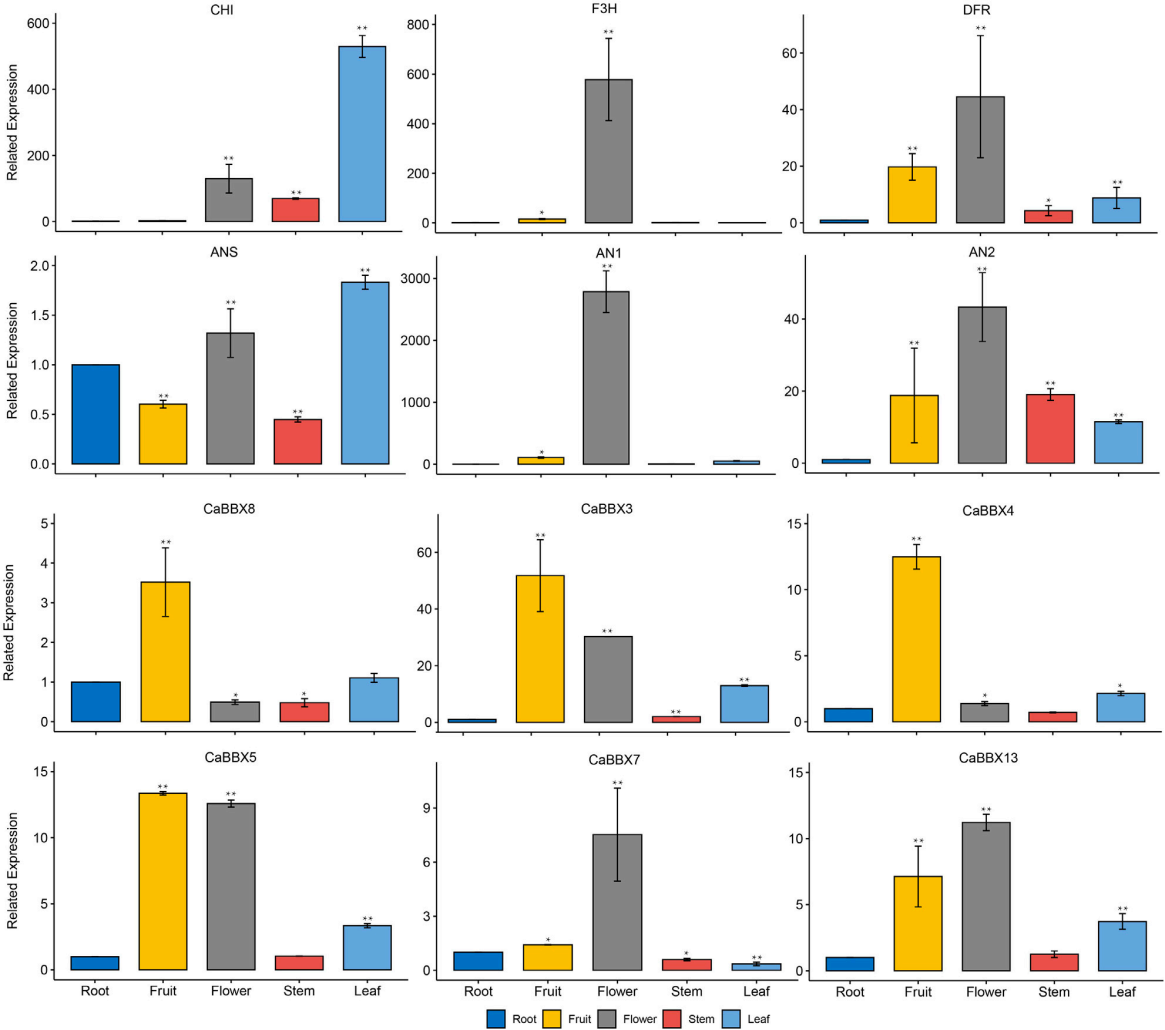
qRT-PCR analysis of *CaBBXs* and the structural genes of anthocyanin biosynthesis pathway. Mean values and standard deviations (SDs) are indicated by error bars. Significant differences were determined by *t*-test (*p < 0.05, **p < 0.01, ***p < 0.001).

Additionally, some typical CaBBXs were screened by qRT-PCR (Real-time polymerase chain reaction) and verification in the cultivated pepper “6421”. Actin gene (GenBank Accession No. Contig0023^*+^) was the endogenous control. Expressions of CaBBX3, CaBBX4, CaBBX5, CaBBX7, CaBBX8, and CaBBX13 were analyzed, which suggested that the qRT-PCR and RNA-seq expression level findings for CaBBXs were similar. Among them, CaBBX3, CaBBX4, and CaBBX8 were highly expressed in fruits but showed lower expression in flowers. Conversely, CaBBX7, and CaBBX13 were highly expressed in the flowers but showed low expression in fruits. CaBBX5 was highly expressed in both flowers and fruits. The CaBBX8, and CaBBX13 were also expressed in other tissues such as leaves and stems. Moreover, expressions of four anthocyanin biosynthesis structural genes (CaF3H, CaANS, CaDFR, and CaDFR) and two typical regulation genes (CaAN1 and CaAN2) were also analyzed in different tissues. The related expressions of CaDFR, CaF3H, CaAN1, and CaAN2 were highly in flowers, while the CaCHI, and CaANS were highly expressed in leaves. The related expressions of CaANS, CaDFR, CaAN2 were also expressed in other tissues such as roots, fruits, and stems (Figure 6). This indicated that CaBBXs may exert important regulatory effects in the anthocyanin pathway during the stages of flowering, pigment accumulation in leaves and stems, and fruit development in pepper.

## Discussion

With the completion of the two germplasm reference genomes, “Zunla” and “CM334”, of pepper (*Capsicum* spp.) in 2014, many families of transcription factors have been identified (Kim et al., 2014; Qin et al., 2014). For example, the WRKY transcription factors (Cheng et al., 2020), HD-ZIP gene family (Zhang et al., 2021), and MYB, one of the largest gene families, have all been characterized by genome-wide studies (Wang et al., 2020b). The B-box (BBX) transcription factors, a subfamily of the zinc finger family of proteins, have several biological functions, which have attracted research attention in recent years (Gangappa and Botto, 2014). In this study, a total of 23 *CaBBX*s were identified in pepper, along with the identification of one more member, *CaBBX18,* in the cultivated species, “Zunla”, absent in the “CM334” reference genome. The *CaBBXs* were unevenly distributed across the 12 chromosomes on the pepper genome and had fewer members relative to *Arabidopsis* (32 *ATBBX*s) (Khanna et al., 2009). Among the Solanaceae plants, the tomato has a much smaller genome than pepper, however, 29 *SlBBXs* have been identified, to date (Chu et al., 2016). Potato contains 30 *StBBX* transcription factors owing to its highly heterozygous tetraploid genome (Talar et al., 2017). Species-specific duplication or deletion during evolution may have led to differences in the numbers of the BBX family of transcription factors across various species.

In pepper, the 23 members were divided into four categories according to the number and types of structural domains, including ten genes with two B-box domains, eight genes with two B-box domains and one CCT domain, four genes with only one B-box domain, and *CaBBX16* gene with one B-box and one CCT domains (Figure 2). The B-box conserved motifs play key roles in protein interaction and transcriptional regulation, while the CCT domain is mainly involved in nuclear transport and transcriptional regulation (Wang et al., 2021). In addition, the exon-intron structures also affected the functions of genes. The number of exons in the *CaBBX* family varied from two to five, and 4.3% of genes in the entire family had no introns. The phylogenetic tree constructed by cluster analysis divided the *CaBBXs* into four clades. Due to the highly conserved exon-intron characteristics, *CaBBXs* in the same clades may have similar functions. Another reason for functional diversity was due to the different *cis*-regulatory elements in the promoter regions (Haberer et al., 2004). A large number of *cis*-elements in the promoter region were related to light responsiveness, including Box 4, G-Box, TCT-motif, and GT1-motif. Many BBX proteins play a key role in light signaling pathways, for instance, *AtBBX20* is a positive regulator of light signaling transduction (Datta et al., 2007). Moreover, ABRE, TC-rich repeats, TCA-element, and ARE involved in responsiveness to abscisic acid or are *cis*-regulatory elements essential for the anaerobic induction. The hormone-related *cis*-regulatory elements, including ERE, CGTCA-motif, TGACG-motif, and GARE-motif were also presented in the *CaBBX* promoter region. Diversified domains, structures, and *cis*-regulatory elements in the promoter region may lead to diverse functions of the *CaBBX* family of transcription factors.

Gene duplication plays a key role in the expansion and functional diversity of gene families (Wang et al., 2020a). A total of three segmental duplicated pairs were found in the inter-species of pepper, a major contributor to gene expansion in the *CaBBX* family. Similar results have been reported in the grapevine *VviBBX* family^29^, tomato *SlBBX* family (Chu et al., 2016), and the pear *PbBBX* family (Cao et al., 2017). No tandem duplications were found. *CaBBX1*-*CaBBX7* and *CaBBX2*-*CaBBX6* were two duplicated gene pairs, and *CaBBX1*-*CaBBX2* were mutual duplicates, in particular. This indicated that *CaBBX1* and *CaBBX2* were duplicated before *CaBBX6* and *CaBBX7*, and *CaBBX6* and *CaBBX7* were in close physical proximity on chromosomes in the genome. The selection pressures were estimated using the Ka/Ks value, and all the three duplicated *CaBBX* pairs had undergone purifying selection (Ka/Ks < 1.0). In addition, these four genes contained two conserved B-box domains, however, their gene structures were different. Both *CaBBX1* and *CaBBX7* contained five CDS regions, while *CaBBX2* had three introns, and *CaBBX6* had only two exons. Although these were duplicated genes, which may arise from the same ancestor, the functions could have diverged due to structural differentiation. Bioinformatic prediction of subcellular localization showed that the *CaBBX1*, *CaBBX2,* and *CaBBX6* genes were localized in the nucleus, while *CaBBX7* had extracellular localization. The localization of *CaBBX7* may support its ability to transport across membranes, however, the specific functions of the *CaBBXs* need further experimental proof. Simultaneously, nuclear localization of the *BBX* family of genes has been reported in other species (Feng et al., 2021). The homologous genes of the duplicated genes *CaBBX1, CaBBX2, CaBBX6,* and *CaBBX7,* belonging to the *CaBBX gene* family are involved in various signaling pathways and development processes. However, differential tissue-specific expression patterns are observed between duplicated gene pairs (Crocco and Botto, 2013). These four duplicated genes were in Clade II in the multi-species phylogenetic tree, which represented the characteristics of the *CaBBX gene* family. The RNA-seq data indicated that *CaBBX1*, *CaBBX6,* and *CaBBX7* were specifically expressed in flowers, while *CaBBX2* was specifically expressed in pepper seeds. Although there was lesser gene duplication in the pepper BBX family, the duplication had no direct effects on the functions of the genes. Through evolution, BBX duplicated genes have gained extensive differences in their structures, motifs, and promoter *cis*-acting elements, which directly contribute to the divergence in the expressions of duplicated genes.

Several studies show that BBX genes are widely involved in the growth and development of plants. For example, the B-box protein, STH2, in *Arabidopsis* is a positive regulator of photomorphogenesis and plays a direct role in transcriptional activation (Datta et al., 2007). Silencing *PpBBX16* reduces the accumulation of anthocyanins and participates in the regulation of anthocyanin accumulation in pear (*Pyrus pyrifolia*) (Bai et al., 2019). *ATBBX18* weakens the tolerance to high temperature in *Arabidopsis* (Wang et al., 2013), and *MdBBX10* enhances tolerance to ABA-mediated responses and ROS levels in apple plants (*Malus domestica* Borkh.) (Liu et al., 2019). However, to date, the specific roles of the BBX family of genes in pepper remain unclear. In this study, RNA-seq data from pepper seeds, flowers, leaves, fruits, and placenta at different stages were used to determine the expression profiles of the *CaBBX* family of genes. All 23 *CaBBXs* were involved in the regulation of fruit development and maturation in pepper, and different genes showed varying tissue-specific expression patterns. Among them, *CaBBX14*, *CaBBX15*, *CaBBX16*, *CaBBX17*, and *CaBBX9* were specifically expressed in the leaves and may be involved in the regulation of light signaling transduction and other pathways. *CaBBX6*, *CaBBX18*, *CaBBX20*, *CaBBX7*, *CaBBX21*, *CaBBX23*, *CaBBX1*, and *CaBBX13* were highly expressed in flowers. *SlBBX20*, the tomato homologous gene of *CaBBX6 is highly* expressed in flowers and is a key regulator of carotenoid biosynthesis (Xiong et al., 2019). The homologous gene of *CaBBX1* and *CaBBX7, the ATBBX19* transcriptional regulator in *Arabidopsis* containing B-box domains, is involved in photomorphogenesis and flowering (Wang et al., 2014). In addition, *CaBBX2* and *CaBBX8* have a seed-specific expression in different organs, and the homologous gene of *CaBBX8, ABBX3,* produces a photoperiodic flowering switch in *Brassicaceae* (Simon et al., 2015). COP1-mediated degradation of the *ATBBX22* gene optimizes seedling development in *Arabidopsis* (Chang et al., 2011). The remaining *CaBBXs* were widely expressed specifically in the placenta or fruits of pepper. *CaBBX10* was only expressed in fruit. Its homologous gene, *ABBX21* of *Arabidopsis,* can interact with COP1 to regulate shade avoidance and long hypocotyl formation (Crocco et al., 2010). The *CaBBX11* and *CaBBX19* were under-expressed in leaves but over-expressed in other organs. While *ABBX28*, the homologous gene of *CaBBX28* encodes an atypical B-box domain protein that negatively regulates photomorphogenic development by interfering with the binding of the transcription factor, HY5, to its target gene promoters (Liu et al., 2020c). In this study, GO enrichment analysis indicated that *CaBBX*s of pepper were widely expressed and enriched spanning across all the stages of fruit development, and their functions were closely related to the differences in their domains, structures, promoters, and evolutionary processes.

In particular, eight family members were specifically expressed in flowers, and eight were specifically expressed in fruits, accounting for 69.56% of the total expression (Figure 4). Specific expression in different organs was considered a significant regulatory mechanism of flavonoid synthesis, growth and development, and fertility stages. In the co-expression network, the *CaBBXs* and anthocyanin structural genes were significantly associated. Seven *CaBBX* genes were closely co-expressed with anthocyanin structural genes (Figure 5). It was speculated that the *CaBBX* transcription factors in pepper play regulatory roles in the processes of anthocyanins biosynthesis in pepper. In other species, some BBXs were involved in regulating the synthesis of anthocyanins, such as the *BBX33/CONSTANS-like 11* (*COL11*) gene which controls the expression of *MYB10* in apple fruit, thereby affecting the synthesis of anthocyanins and regulating the color of the red skin (Plunkett et al., 2019). The overexpression of *BBX23* in poplars (*Populus trichocarpa*) activates the expression of MYB transcription factors and structural genes in the flavonoid pathway, thereby promoting the accumulation of procyanidins and anthocyanins (Li et al., 2021). In this study, the *CaBBX* gene family was systematically studied by both RNA-seq and qRT-PCR. *CaBBX3*, *CaBBX4*, and *CaBBX5* were identified as three candidate anthocyanins regulatory genes in fruits, while the *CaBBX6*, *CaBBX7*, and *CaBBX13* were three candidate anthocyanins regulatory genes in flowers. However, the specific regulatory mechanism and function of these candidate genes need to be verified by further molecular biology experiments. This study provided a strong reference for screening the transcription factors involved in the regulation of anthocyanin synthesis in pepper and laid a solid foundation for the further functional characterization of the *CaBBXs*.

## Data Availability

The original contributions presented in the study are included in the article/Supplementary Material, further inquiries can be directed to the corresponding authors.
