# Supplementary figures and images for: Genome-Wide Characterization and Anthocyanin-Related Expression Analysis of the B-BOX Gene Family in Capsicum annuum L
Source: Front Genet. 2022 Feb 28;13:847328. doi: 10.3389/fgene.2022.847328 (PMC8918674; doi:10.3389/fgene.2022.847328)

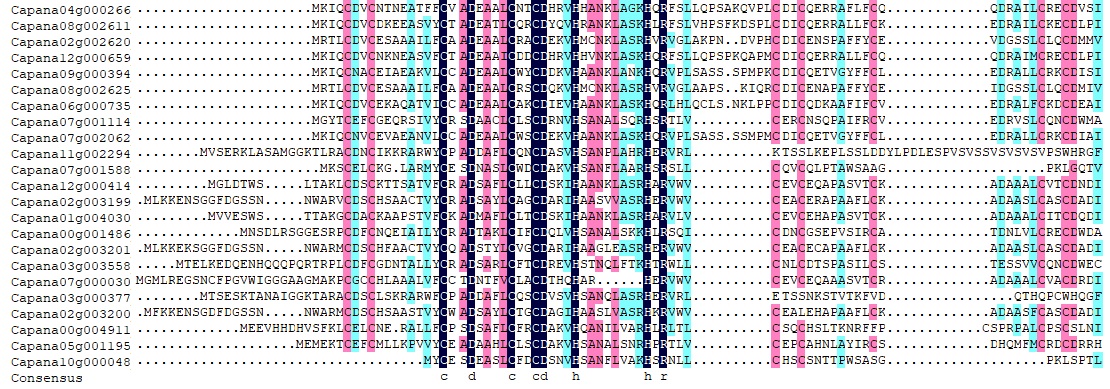

Supplement: Supplementary file 1 [file Image1.JPEG]

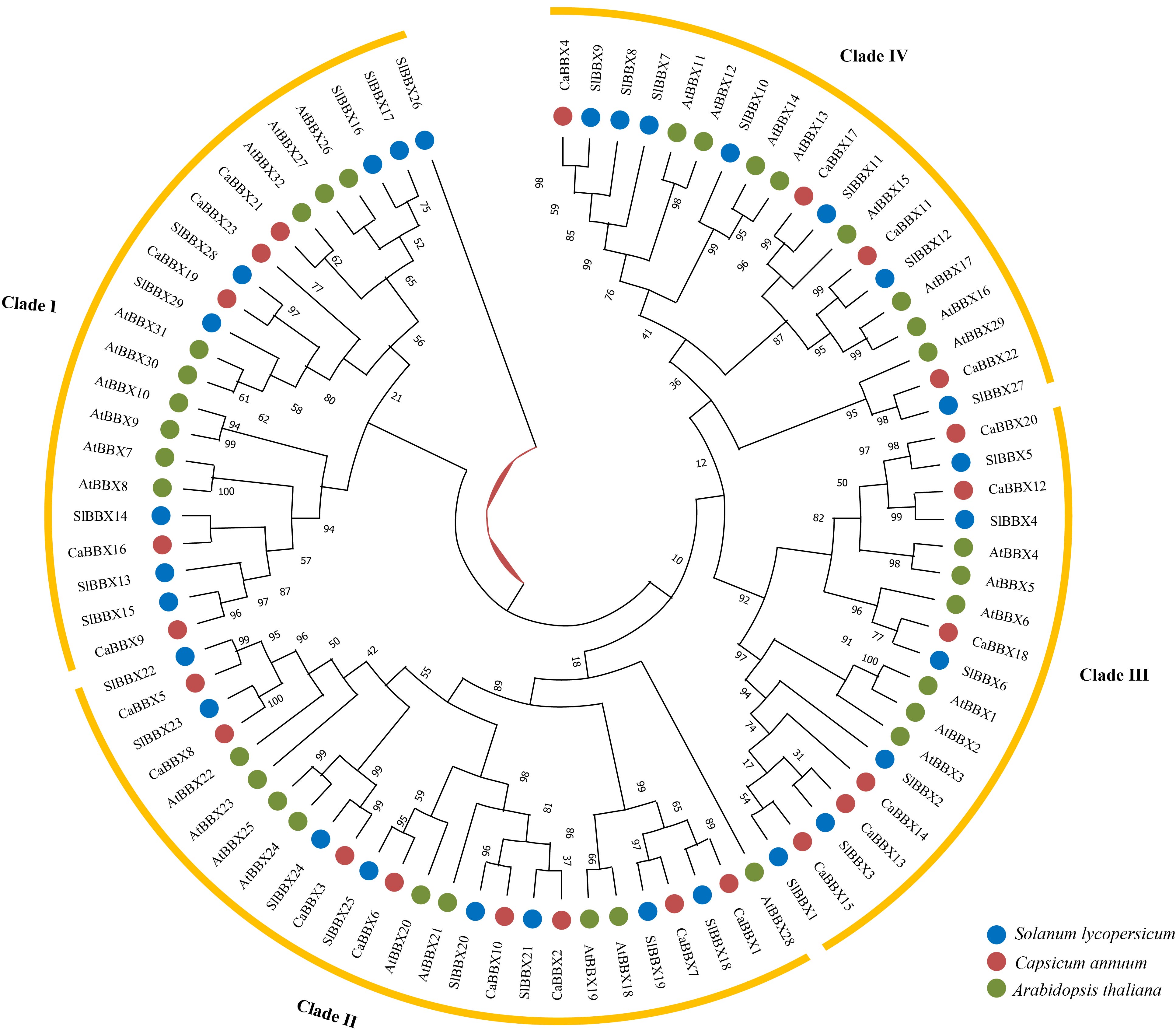

Supplement: Supplementary file 2 [file Image2.JPEG]
