# Supplementary material for: Genome-Wide Characterization and Anthocyanin-Related Expression Analysis of the B-BOX Gene Family in Capsicum annuum L
Source: Front Genet. 2022 Feb 28;13:847328. doi: 10.3389/fgene.2022.847328 (PMC8918674; doi:10.3389/fgene.2022.847328)
Supplement: Supplementary file 3 [file Table2.DOCX]

Table S2. Primers for qRT-PCR

| Marker name | GeneID | Primer sequence (5’–3’) | Annealing |
| --- | --- | --- | --- |
|  |  |  | temp (°C) |
| *CaCHI* | *Capana00g002736* | F AACACCACCTTGTTCCTTGC | 58 |
|  |  | R CTTGGAGCTTTTCCCTTTCC |  |
| *CaF3H* | *Capana02g002586* | F CGGATCCCCGCACGGATGGATAGCTG | 57.8 |
|  |  | R CGGATCCGGTTAAGGCCTCCTTCTCC |  |
| *CaDFR* | *Capana02g002763* | F AACGCTGTGGAAAGCAGACT | 59 |
|  |  | R AGGGTCCTTGGACTCGAAAT |  |
| *CaANS* | *Capana10g001654* | F TGAGGGATAAGGAGCATTCG | 60.5 |
|  |  | R GCATTGCCGGAGACCTAATA |  |
| *CaAN1* | *Capana00g003336* | F AAAGTGGCACCTTGTTCCTG | 59 |
|  |  | R ATTCAACCACCGAAGTCTGC |  |
| *CaAN2* | *Capana10g001433* | F GCTCACAAGTGAAACGTCGT | 58.5 |
|  |  | R AAAGATCCCAACTGCAGCCA |  |
| β-actin | AY572427 | F AATCAATCCCTCCACCTCTTCACTC  R CATCACCAGCAAATCCAGCCTT | 61 |
| *CaBBX3* | *Capana06g000735* | F TGGTTGGGAGGCATTGATCT R GTGCCGATAGCTCAGGTACT | 60.5 |
| *CaBBX4* | *Capana07g001114* | F TGATGCAGCTTGCTTGTGTT  R GGAATGACGCTGTGACAAGG | 59 |
| *CaBBX5* | *Capana07g002062* | F TAGACGGTGATGAGTGCT  R GGCCAGTAAAGTCCAGAG | 58.2 |
| *CaBBX7* | *Capana08g002625* | F GGCCCTTCAAATGAGCTA  R TGCCATAGCAATCTCCCT | 59.5 |
| *CaBBX8* | *Capana09g000394* | F CTTGCTGACCGGAGTCAAAG  R TTCTGAATGGGAGGCGACTT | 55.9 |
| *CaBBX13* | *Capana02g003199* | F CTGACATCCATTCTGCAA  R TAGAACCACCGCCAACAG | 60 |
